# Bladder cancer: therapeutic challenges and role of 3D cell culture systems in the screening of novel cancer therapeutics

**DOI:** 10.1186/s12935-023-03069-4

**Published:** 2023-10-25

**Authors:** Sameh M. Farouk, Asmaa F. Khafaga, Ahmed M. Abdellatif

**Affiliations:** 1 Department of Cytology & Histology, Faculty of Veterinary Medicine, Suez Canal University, Ismailia, 41522 Egypt; 2 Department of Pathology, Faculty of Veterinary Medicine, Alexandria University, Edfina, 22758 Egypt; 3 Department of Anatomy and Embryology, Faculty of Veterinary Medicine, Mansoura University, Mansoura, Egypt

**Keywords:** Bladder Cancer, 3D cell culture, Chemo-resistance, Cancer Stem cells, Drug Resistance, Drug Discovery

## Abstract

Bladder cancer (BC) is the sixth most common worldwide urologic malignancy associated with elevated morbidity and mortality rates if not well treated. The muscle-invasive form of BC develops in about 25% of patients. Moreover, according to estimates, 50% of patients with invasive BC experience fatal metastatic relapses. Currently, resistance to drug-based therapy is the major tumble to BC treatment. The three-dimensional (3D) cell cultures are clearly more relevant not only as a novel evolving gadget in drug screening but also as a bearable therapeutic for different diseases. In this review, various subtypes of BC and mechanisms of drug resistance to the commonly used anticancer therapies are discussed. We also summarize the key lineaments of the latest cell-based assays utilizing 3D cell culture systems and their impact on understanding the pathophysiology of BC. Such knowledge could ultimately help to address the most efficient BC treatment.

## Introduction

Bladder cancer (BC) is among the top 10 cancers affecting men [1]. The incidence of BC is higher in men than in women [2, 3]. About 25% of BC cases proceed to the muscle-invasive form. Lethal metastatic relapses are estimated to occur in 50% of patients with invasive malignancies [4]. The rates of metastatic BC increase with age and are seen most frequently in elderly people [5]. The risk factors for BC include smoking, inflammation, exposure to carcinogens, and schistosomiasis [6]. BC typically starts inside the bladder epithelium and travels from there to muscles and other tissues [7]. Over 90% of urothelial carcinomas were reported to originate in the urinary bladder [8]. The most common cause of death in advanced BC patients is associated with metastasis of BC [9]. Hematuria represents the most characteristic symptom of BC and is usually diagnosed by physical inspection of the urine [10]. However, patients can also present with isolated microscopic hematuria which is often detected during routine cystoscopic examinations [11].

Cell culture is an important technique for maintaining cells outside the body. Under optimal conditions, the appearance of cultured cells or organoids can be used to reflect their in vivo behavior. The alignment of cells in the culture vessel significantly affects their structure, and functions, as well as their response to the tested chemical compounds [12]. Three-dimensional (3D) models of cell culture have emerged and are currently representing a useful platform for monitoring cellular organization via comprehensive visualization of the cultured cells [13]. Since the function and morphology of individual cells substantially rely on their interactions with proteins and signaling factors from neighboring cells and their surrounding extracellular matrix, the efforts for culturing cells in 3D systems have progressively evolved [14]. Culturing cells in 3D systems has been suggested to recover some of their natural characteristics that are usually affected during their culture in conventional, two-dimensional (2D), culture systems [15]. Recovering the cellular characteristics of cultured cancer cells could help for increasing both the specificity and sensitivity of cell-based assays used for determining their identities and also for addressing and selecting new drugs for cancer treatment [16].

## Classification of bladder cancer (BC)

**Fig. 1:**
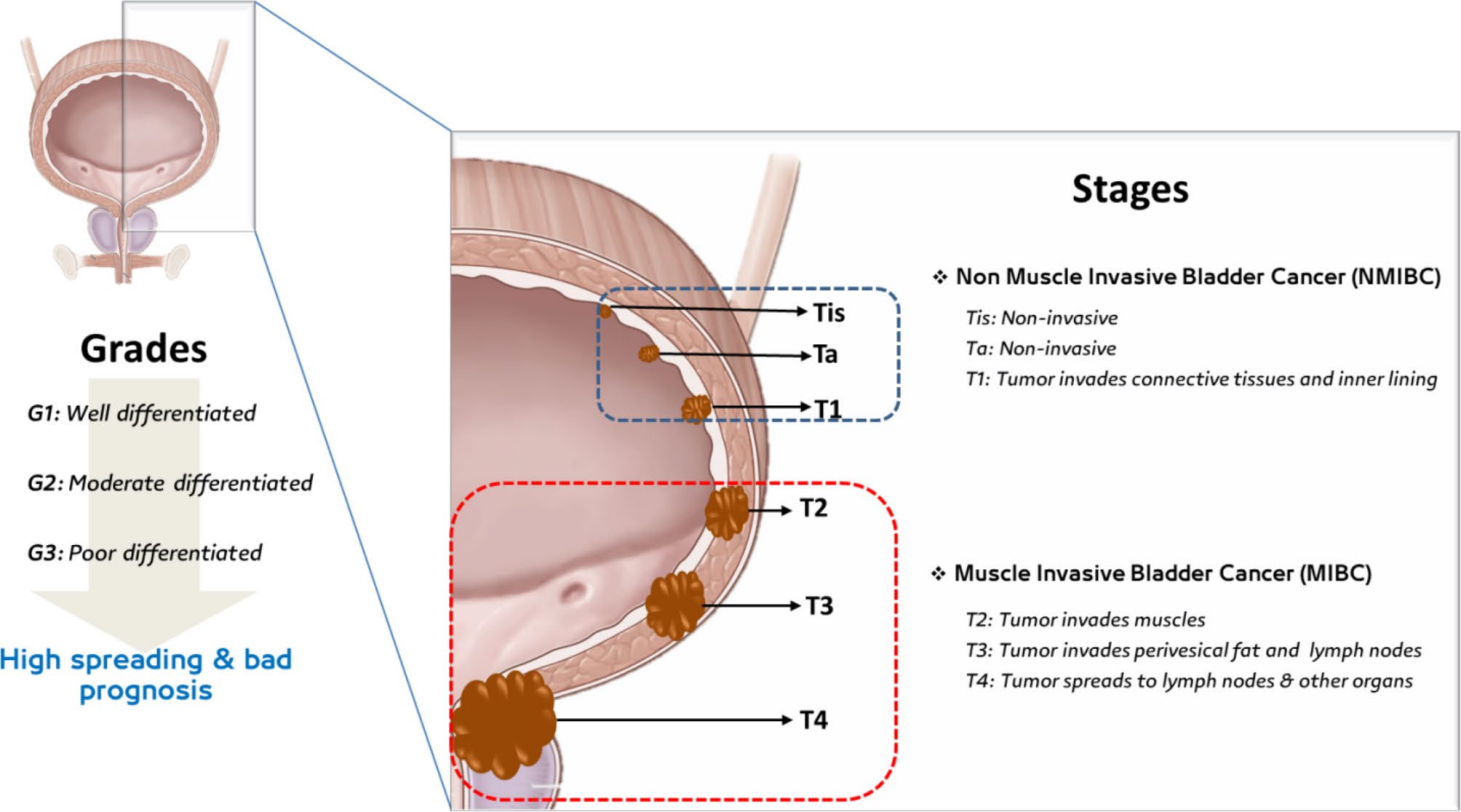
Grades and stages of bladder cancer

Understanding the classification of BC is important to establish an appropriate treatment strategy. The World Health Organization (WHO) initially classified BC into three grades: well-differentiated (grade 1 or G1), moderately differentiated (grade 2 or G2), and poorly differentiated (grade 3 or G3) papillary urothelial carcinoma (PUC) [17]. The latter-mentioned grade is the highest grade, in which the poorly differentiated cancer cells grow at faster rates and start to spread to other organs including the regional lymph nodes. Urothelial carcinoma, squamous cell carcinoma, and adenocarcinoma are the three most common microscopic subtypes of BC [7]. Based on the progressive and invasive nature of the continuously proliferating tumorous cells, BC is classified into two main stages: non-muscle invasive bladder cancer (NMIBC) and muscle-invasive bladder cancer (MIBC) [18, 19]. NMIBC represents roughly 70–85% of BC cases in which patients have tumors restricted to the mucosa-submucosa layers, hence it is superficial BC [20]. It is further classified into Tis, Ta, and T1. In Tis and Ta, cancer is restricted to the urothelial layer, while in T1 it reaches to the underlying connective tissue layer. Similarly, MIBC is further categorized into the degree of cancer invasion into T2 (muscle layer), T3 (perivesical fat and lymph nodes), and T4 (other organs). Various grades and stages of BC are illustrated in Fig. 1.

## Strategies for treatment and management of bladder cancer (BC)

Proper BC diagnosis is essential for selecting a specific treatment. Urine cytology and cystoscopy are the most widely significant tools for BC diagnosis and follow-up [20]. Although cystoscopy remains as an essential investigative gadget in the disclosure and monitoring of BC, small papillary tumors or carcinoma in situ can be easily omitted by standard white-light cystoscopy (SWLC), which may purpose for early recurrence of the disease. This leads to the development of novel diagnostic technologies such as narrow-band imaging cystoscopy and photodynamic technology [21]. Various molecular urinary tests have been marketed over the years to help in the detection of BC. Although initially hopeful, none of the different technologies has been enough specific or sensitive to prohibit cystoscopic surveillance [22, 23].

Neoteric advances in BC management are increasing. These advances include the use of cystoscopic and fluoroscopic revelation, neoadjuvant chemotherapy, bladder-sparing multimodal therapy, and intravesical therapy [24]. As mentioned earlier, most BC cases do not encompass the bladder muscular wall and are commonly handled with telescopic removal of cancer (transurethral resection of bladder tumor), followed by infiltration of vaccine-based therapy or chemotherapy into the bladder [25].

## Resistance of bladder cancer (BC) to chemotherapy

Chemo-resistance is one of the main problems in treatment of various types of cancer as cancer cells become resistant to chemotherapeutic agents [26]. Recurrence of cancer is a serious trouble in patients with BC with increased proliferation rates of drug-resistant cells [27]. The persistence of cancer cells` resistance to chemotherapeutics is a major stumbling to BC treatment [28]. Among the causes that BC is so deadly is its tendency to develop drug resistance typically used as frontline therapies [29]. Some cancers are considered resistant to therapy, either innate drug resistance at the time of drug exposure or acquired drug resistance after an initial response [30]. Although BC is a chemotherapy-sensitive malignancy, nearly most of patients promote disease progression after an initial chemotherapeutic response [31].

Radiotherapy has been suggested as a promising technique for control of muscle-invasive form of BC [32, 33]. Cryotherapy -also called cryoablation or cryosurgery- involves heat extraction from cancer cells via application of extreme cold (< 0° C) which will eventually lead to death of the cancerous cells [34]. Percutaneous cryotherapy revealed a decline in the incidence of complications associated with BC including hematuria and urinary irritations [35].

Transurethral resection of bladder cancer (TURB) is frequently used for management of non-muscle invasive BC with about 50% rate 5-year overall survival [36, 37]. However, this rate declines to 20% by 15 years following tumor resection [36].

The use of multimodal techniques showed better outcomes for treatment of BC than single intervention [38]. For instance, decreased cancer survival was seen in BC patients subjected to cryoablation combined with the chemotherapeutic agent cisplatin compared to those who received cisplatin only [39]. In addition, patients who underwent TURB followed by radiochemotherapy displayed a higher rate of BC remission and longer overall survival than those who received radiotherapy alone [40].

## Drug resistance mechanisms in bladder cancer (BC)

**Fig. 2:**
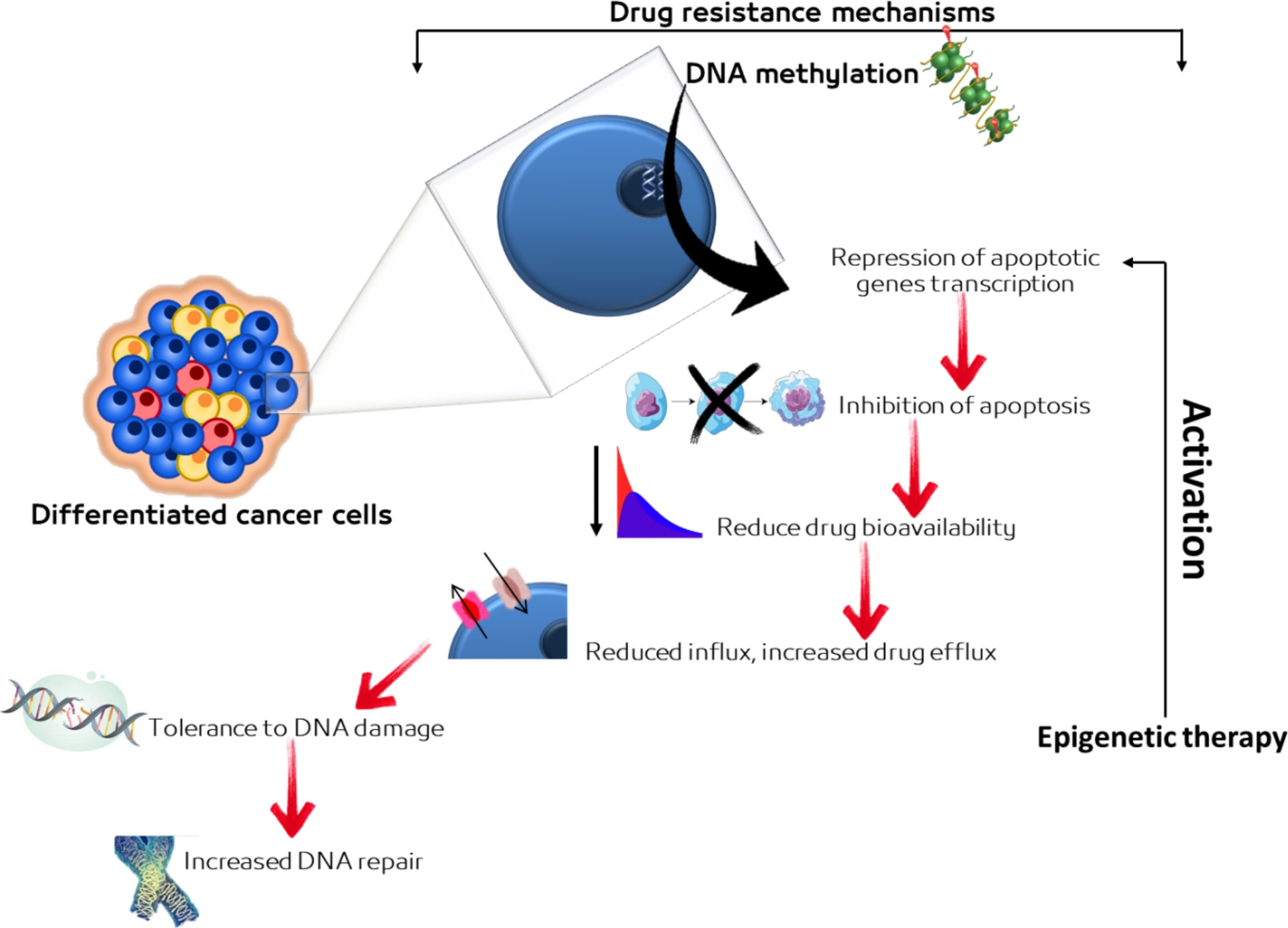
Possible mechanisms of drug resistance in bladder cancer. The diagram was created with BioRender [45]

Drug resistance in BC comprises numerous mechanisms, such as avoidance of apoptosis by cancer cells via DNA methylation-induced transcriptional repression of genes participating in the apoptotic pathway [27]. Additionally, activation of these genes by epigenetic therapy might expedite the reconquest of chemotherapeutic agents’ sensitivity in BC and could lead to novel therapeutic approaches in BC [31]. Drayton and Catto [41] stated that the mechanisms of drug resistance could be classified into these act to weaken the normal cellular response to drug-induced DNA damage and those act to reduce drug bioavailability within a cell. Reduced influx, increased drug efflux, increased DNA repair, and tolerance to DNA damage appear to be the preponderant mechanisms of drug resistance [28]. The mechanisms related to radio- and chemo-resistance influence many pathways as those involved in DNA damage repair, drug absorption and efflux, cell cycle, and apoptosis [29]. The use of phytochemicals has shown promising effects in mitigation of drug resistance of cell lines and animal models of BC [42, 43], though their implementation in clinical protocols remains under deep investigation [44]. The possible mechanisms of drug resistance in BC are summarized in Fig. 2.

## Drug Resistance regulation by bladder cancer (BC) stem cells

Cancer stem cells (CSCs), also known as tumor-initiating cells (TICs), have been intensively studied in the past decade, with a focus on their origin, possible sources, cellular markers, survival mechanisms, and development of therapeutic strategies targeting them [46, 47]. CSCs have been proposed to play a major role in tumorigenesis, drug resistance, metastasis, and cancer relapse, because of their ability for self-renewal [48]. The subpopulation of CSCs that remains in tumor tissue following chemotherapy is responsible for survival and expansion of tumor cells during recurrence [49, 50].

**Fig. 3:**
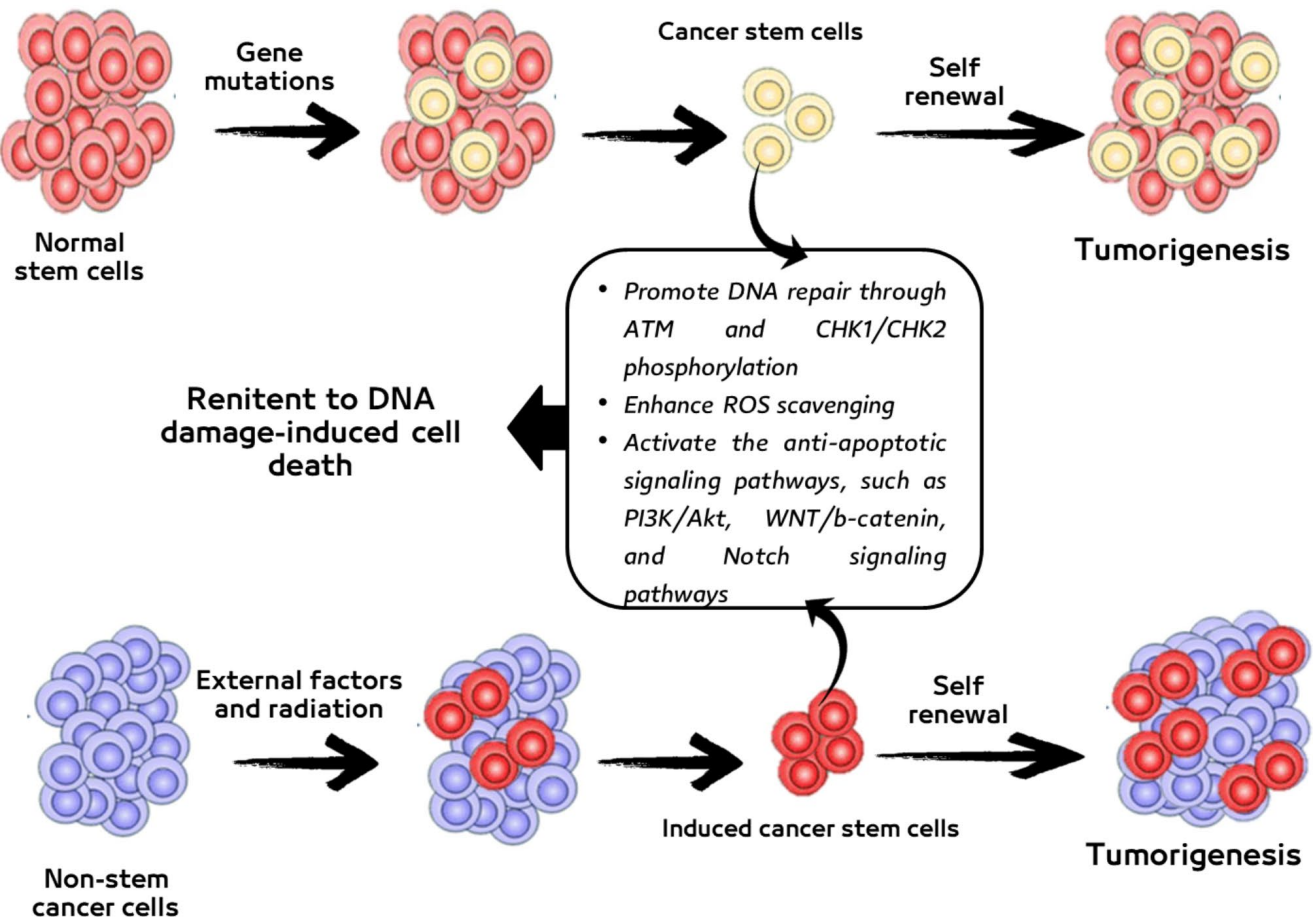
Different sources of cancer stem cells (CSCs) and their possible role in tumorigenesis and inhibition of DNA damage-induced cell death. The diagram was created with BioRender [45]

CSCs can be renitent to DNA damage-induced cell death through different ways. These ways include protection against oxidative DNA damage by enhanced ROS scavenging, promotion of the DNA repair capability through ATM and CHK1/CHK2 phosphorylation, or activation of the anti-apoptotic signaling pathways, such as PI3K/Akt, WNT/b-catenin, and Notch signaling pathways [47, 50] **(**Fig. 3**).** For instance, CD44 interacts with a glutamate-cystine transporter and controls the intracellular level of reduced glutathione; hence, the CSCs expressing a high level of CD44 showed an enhanced capacity for GSH synthesis, resulting in stronger defense against ROS [51].

It is widely supposed that CSCs may emerge from normal stem cells that have sustained gene mutations [52]. CSCs can also be constructed from differentiated or progenitor cells that undergo de-differentiation or tumor cells that acquire stem cell properties [53, 54]. It has been noted that BC stem cells (BCSCs) originated from CSCs or from BC non-stem cells (BCNSCs) with clonal identity [55, 56] **(**Fig. 3**).**

Several common markers of BCSCs, including CD44+, BCMab1+, EMA-, and 67LR+, are expressed in the basal cell layer of BC mass that leading to more debates regarding the exporter of BCSCs [57, 58]. Theoretically, if all markers are from a specific cell type in BC, it is supposed that BCSCs may have arisen from mutated normal stem cells. On the other hand, if the markers are expressed on different normal cell types, then the BCSCs may be derived from differentiated or progenitor cells that acquired de-differentiation characteristics due to mutations, thus leading to different BCSCs subgroups [59].

Many types of cancers are associated with autocrine signaling of different cytokines that are capable of activating receptors largely falling under the receptor of tyrosine kinase family [60, 61]. Vascular endothelial growth factor (VEGF), hepatocyte growth factor (HGF), and stem cell factor (SCF) are a few important autocrine players that could mediate the maintenance of BCSCs since their receptors were exposed in BC cell lines [62].

Vascular endothelial growth factor receptor 2 (VEGFR2) is implicated in CSCs that can trigger blebbishield emergency program-mediated sphere formation in RT4 (transitional cell papilloma) bladder cancer cells [63]. cMET and cKit receptors were co-downregulated along with VEGFR2 during blebbishield formation, indicating that these receptors might be activated along with VEGFR2 since receptors usually undergo downregulation after activation [63, 64]. VEGFR2 is usually detected in endothelial cells, and endothelial cells are known to create stem cell niches implicating endothelial cells in tumors as one of the culprits that could transform CCs into CSCs [65]. The presence of cMET, VEGFR2, and cKIT also might help bladder cancer stem cells to mediate metastasis [62].

## Three-dimensional (3D) cell culture system for studying drug resistance in bladder cancer (BC)

3D cell culture systems are becoming incrementally popular in contemporaneous cancer studies, tissue engineering, drug discovery, and drug resistance research because of their obvious advantages in providing more predictive data for in vivo tests and more physiologically relevant information [66, 67].

The conventional 2D culture systems involve culturing the targeted cells in a monolayered structure either inside a flat petri dish or a culture flask [68]. The main advantages of these culture systems include low-cost and simple maintenance of the cell culture. On the other hand, 2D culture systems have several disadvantages. The most important disadvantage is that the 2D cultured cells do not mimic the natural morphology and behavior of normal tumor cells. Another disadvantage of the 2D culture systems involves the improper interactions between cultured cells as well as between the cells and their surrounding matrix. Lack of such interactions is suggested to adversely affect the growth, vitality, and differentiation of cancer cells. The latter effects have been linked to abnormal gene expression and lowered drug metabolism and responsiveness [69–71]. Indeed, altered morphology and mode of division of cancer cells were noted following their transfer from original tissues into 2D cultures [72, 73]. These alterations in cancer cell morphology affect their secretory and signaling activities [74–77]. The lack of sufficient contact between cells and extracellular matrix is associated with loss of cell polarity [78]. The latter modifies the cell response to damaging stimuli including apoptosis and other associated phenomena [79, 80].

Another important disadvantage of 2D culture systems is that the monolayered cells have great access to the culture medium that consists principally of essential nutrients, metabolites, and oxygen. Due to the natural architecture of the tumor cells within the solid tumor mass, the in vivo cancer cells display variable access to oxygen and nutrients [69]. Importantly, the 2D cultures allow the study of cell type only [81], this leads to marked lack of data about tumor microenvironment, which is required in vivo by cancer-initiating cells [82, 83]. Those disadvantages of 2D systems drove scientists and investigators to find alternative culture models able to mimic the natural structure and morphology of tumor cells.

In 3D culture systems, striking parallels between the morphology and behavior of cells expanding in a tumor mass and cells cultivated in a 3D environment have been thoroughly characterized and verified [69, 84]. The idea of 3D spheres is based on the construction of multilayered spheroid structures: the physical and metabolic characteristics of a solid tumor mass are thus mimicked. Around 40 tumor cell lines were morphologically analyzed and cultured in 3D spheroid conditions. These cell lines came from glioblastoma, astrocytoma, Wilms’ tumor, neuroblastoma, head and neck squamous cell carcinoma, melanoma, lung, breast, colon, prostate, ovarian, hepatocellular, and pancreatic cancers. Based on the architecture of spheroids, three distinct groups were identified: (1) tight spheroids, (2) compact aggregates, and (3) loose aggregates [85, 86].

The 3D models provide appropriate cell-cell and cell-environment interactions, which were built in order to get an imitation of tissue structure. As occurs in vivo [87, 88], cells can be stimulated by their immediate surroundings. Additionally, in 3D cultures, the morphology and polarity of the cells are well-preserved and can be changed back to those of cells that were previously cultivated in 2D [89]. Similarities between 3D culture and cells growing in vivo in terms of cellular topology, gene expression, signaling, and metabolism are another significant feature [90–95]. Tumor drug resistance appears to be significantly influenced by interactions between cells and the extracellular matrix (ECM). A good technique to replicate the organic structure of a tumor mass is to employ synthetic ECM [96]. In this regard, the use of 3D systems could prevent the over- or underestimation of a particular medicine in the case of drug sensitivity and resistance, as well as its dosage [87, 97].

A negative side of 3D culturing is that single cells must be removed from the spheroid by proteolytic breakdown of single layers, which can take up to a few days [98]. Moreover, data repeatability and worker comfort are frequently more challenging in 3D approaches than in 2D systems [99]. The fact that “spheres” can be constructed from a few cell clusters rather than a single cell is frequently cited as a drawback of 3D structures. However, even structures made from a collection of cells retain a three-dimensional form more accurately than adherent, flat cultures [100].

Tumor masses are made up of tumor cells with a variety of phenotypes rather than being a homogeneous structure. In addition, several cell phenotypes are combined in 2D cultures as well. However, by cultivating a single cell with a single genetic background in a concentrated culture medium, such as soft agar or Matrigel, a homogeneous structure can be produced [101]. Vinci and colleagues’ description of a three-dimensional spheroid-based functional assay for cancer target validation and medication evaluation provided a solution to the problem of low reproducibility in 3D culture. Each well on the 96-well ultra-low attachment plates contained a single spheroid. The resultant spheroids’ sizes were consistent and had a Gaussian (normal) distribution [85, 86].

**Fig. 4:**
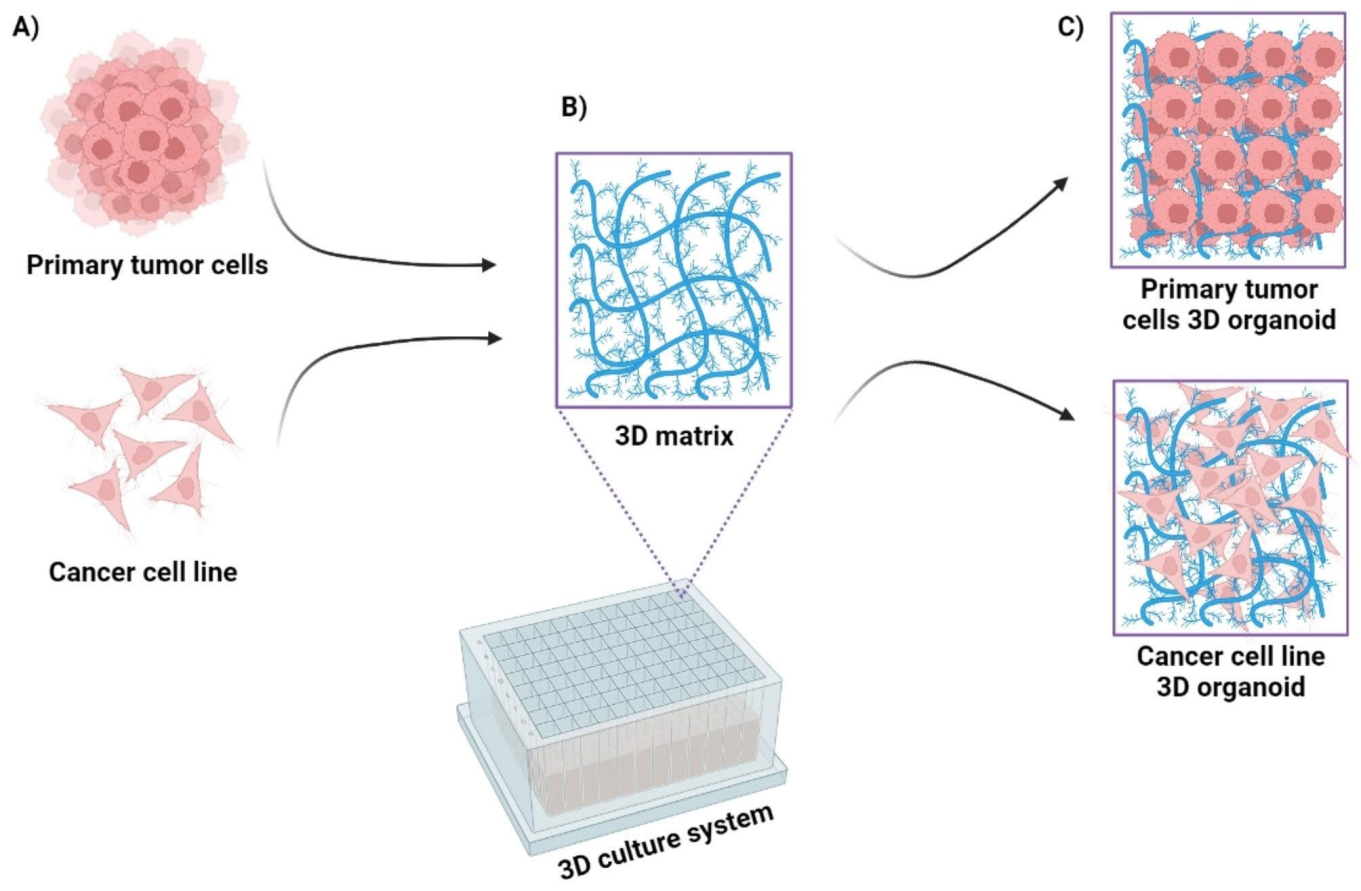
Summary of the main steps used for the preparation of three-dimensional (3D) culture models of cancer cells. **(A)** Dissociated cells are collected from primary tumors or cancer cell lines. **(B)** Obtained cancer cells are then incubated with an appropriate extracellular matrix, e.g., collagen, to ensure their 3D orientation. **(C)** A 3D organoid is formed. The diagram was created with BioRender [45]

Detailed protocols for generation of 3D organoids from murine and human BC cells are currently available [102, 103]. The principal steps in generation of these 3D cancer models are simplified in Fig. 4.

**Fig. 5:**
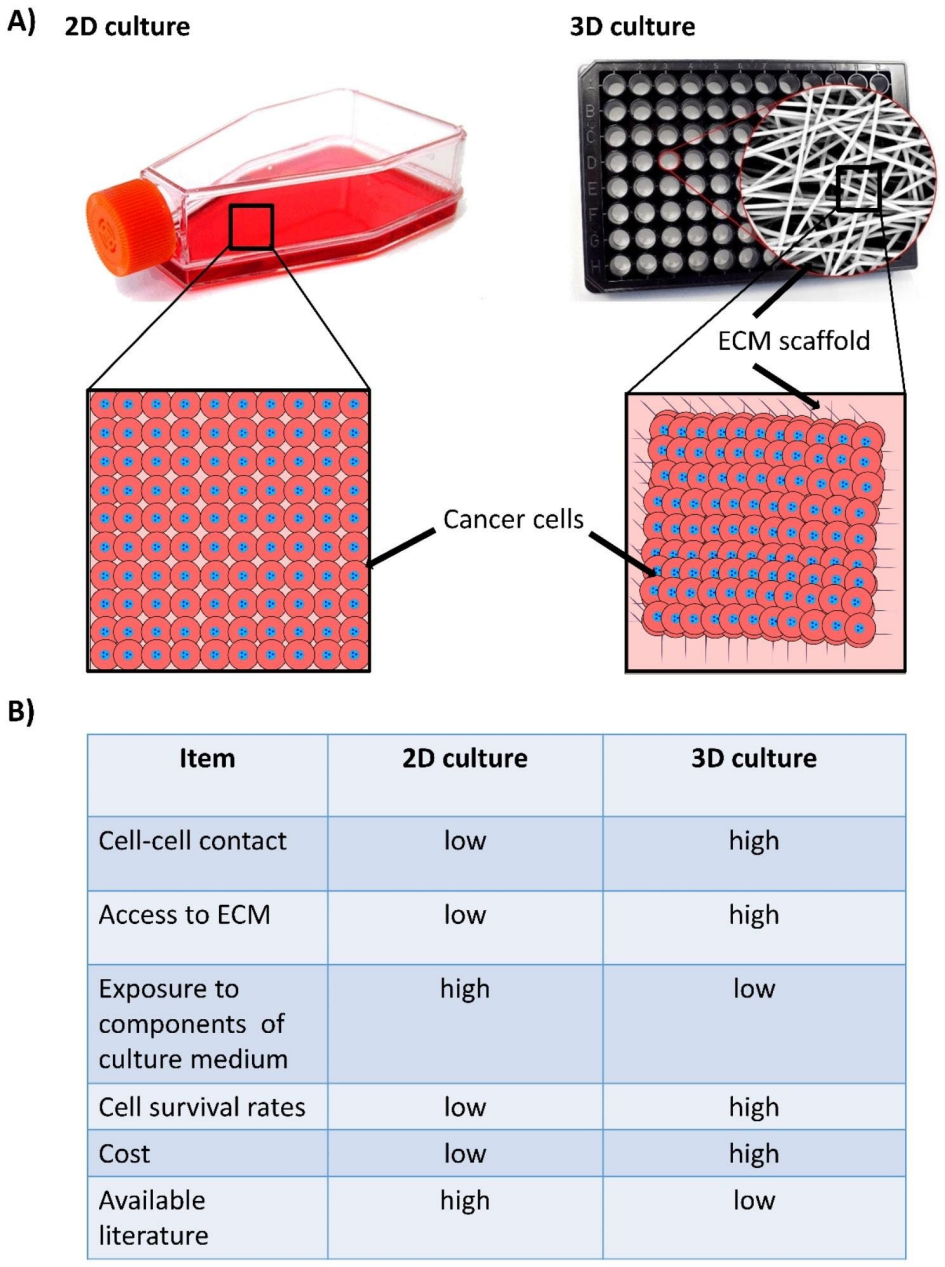
Major differences between two-dimensional (2D) and three-dimensional (3D) culture systems of cancer cells. **(A)** Alignments of cells within the culture vessel. **(B)** Tabulated summary of the advantages and disadvantages of each system. ECM, extracellular matrix

Due to the numerous issues associated with 2D systems, 3D models would seem to be an excellent substitute that might serve as a bridge between 2D and animal studies [104, 105]. There are benefits and drawbacks to the various technical methods for creating 3D models. The choice of 3D system to be used relies mostly on the type of research being conducted. The fact that using the incorrect model can affect the outcomes must be underlined. It is evident that there is no perfect 3D model. It may be sufficient to employ a 2D culture system in some circumstances, but as automation and costs are reduced, the high cost of the technique and the relative scarcity of the available literature remain as two major obstacles facing the widespread use of 3D culture system in BC modeling **(**Fig. 5**).** 3D culture models are believed to be used more frequently in the future.

As CSCs are critical for tumorigenesis, metastasis, tumor growth, and recurrence, there is a need to set up a 3D culture system analogous to the conditions of in vivo tumorigenesis [66]. Concerning metastasis and drug resistance, 3D culture models should better enhance the growth of TICs to mimic the actual tumorigenic processes, creating such appropriate conditions will result in the existence of molecular events relevant to metastasis [106].

3D CSCs culture models may also allow for a better assessment of cellular morphology and cellular proliferation rates. CSCs segregated from several types of in vivo tumors that exhibited prevalent lineaments: relative quiescent state, self-renewal capacity, and mutual tumor–stem‐cell morphological properties; their predilection to develop in a spheroid‐like manner [107]. Growing CSCs in 3D cell culture prompts cell proliferation more rapidly at the periphery of the spheroid because of the lack of cellular molecules adherence. Conversely, this is caused by the loss of CSCs polarity during the process of epithelial-mesenchymal transition, an important step for initiation of cancer metastasis [108, 109]. In addition, stem cell markers percentage is generally high in 3D cell culture models [110].

Selection of the most appropriate cancer therapeutic agent requires thorough in vitro analysis and validation before transitioning to clinical trials. 3D CSCs models mimic tumor microenvironments better, elucidate a more factual drug response, exhibit more adequate proliferation rates with more representative cellular morphology, facilitate the formation of ECM and stimulate high expression of ‘stemness-related’ genes [66].

Regarding culturing 3D tumorspheres, there are two main techniques: scaffold-free techniques, e.g., the hanging drop and suspension method, and scaffold-based techniques, e.g., scaffolds and hydrogels [111]. Both approaches allow for biochemical communication between TICs and the ECM; this interaction is critical for recreating the tumor-tissue microenvironment (TTM) observed in vivo [111, 112]. Amaral et al. recorded that the forced floating method using ULA 96-well round-bottomed plates was considered more reliable to create RT4 spheroids for drug screening/cytotoxicity assays than the hanging drop method [113].

Studies involving the use of 3D organoids in modeling of BC are progressively increasing. Advantageous effects of the 3D BC culture systems over the traditional BC culture systems include an increased rate of cancer cell proliferation and survival and enhanced sensitivity to chemotherapeutic agents. Summarized findings of studies utilized the 3D culture systems for BC modeling [114–122] are listed in Table 1.

**Table 1:** Summary of studies used three-dimensional (3D) culture models in bladder cancer

Study material	Method of 3D culture	Topic investigated	Impact on cancer cell	Reference
3D bio-printed and 2D cell cultures of T24 and 5637 cells	T24 and 5637 were cultured on a synthetic 3D scaffold.	The cell survival rates in the 3D and 2D cultures and sensitivity of cells to rapamycin and Bacillus Calmette-Guérin (BCG)	Cells of 3D cultures demonstrated higher proliferation rates and more exaggerated response to rapamycin and BCG than those of 2D cultures	[114]
Bladder and prostate cancer cell lines	Spheroids were generated from T24 and SV-HUC-1.	The cytotoxic effect of ciprofloxacin and levofloxacin on cell lines during culture	Both drugs exhibited a toxic effect on the tested cell lines (↑ apoptosis; ↓ S phase cell proliferation).	[115]
Tumor cells from BC patients and BC cell lines (RT4, UM-UC-3, and HT1376)	Microtumors were created using a self-assembly process.	The gene expression profiles of cells of the 3D microtumors and those of traditional cultures	A more invasive phenotype was observed in 3D microtumors that was associated with upregulated expression of Delta-like ligand 4 (DLL4)	[116]
Organoids of human BC cell lines and primary cancer cells	Primary cell organoids (BCa #01)	The effect of Wnt/β-catenin pathway activation, using CHIR99021, on cancer cell proliferation	Wnt/β-catenin activation increased proliferation of BC cells grown in 3D cultures but not in conventional adherent systems	[117]
Human urothelial cancer of the bladder (HUCB)	3D co-cultured spheres of HUCB cells and tumor-associated macrophages (TAMs) and cancer-associated fibroblasts (CAFs).	The paracrine effect of TAMs/CAFs on tumor microenvironment	3D co-culture of HUCB cells and TAMs/CAFs increased CXCL1 production in culture with subsequent increase in cell-to-cell interaction among cancer cells and TAMs/CAFs	[118]
3D-spheroids of BC cell lines RT4 and 5637	RT4 and 5637 spheroids were prepared using the aggregation-based method. 2 × 10^5^ or 1,000 cells (respectively) were seeded in 6- or 96-well U-bottom plates coated with poly-HEMA.	Protein expression of the luminal markers peroxisome proliferator activated receptor γ (PPARγ) and forkhead box A1 (FOXA1) in cancer spheroids	PPARγ and FOXA1 proteins were expressed to a lesser extent in cancer spheroids than in cells grown in 2D cultures.	[119]
3D-spheroids of human BC primary cells	Biopsies from bladder tumors were fragmented and allowed to form 3D spheroids.	Sensitivity of the cancer cells to the chemotherapeutic agents mitomycin C, thiotepa, epirubicin, and adriamycin	Mitomycin C achieved the best results with mean sensitivity of 50%, followed by thiotepa (37%), epirubicin (7%), and adriamycin (3%).	[120]
Prostate and bladder cancer cell lines	5637 and T24/TSU-Pr1 cell lines were pelleted and resuspended into 50 mL Bioreactor tubes at density of 100,000 cells/mL.	Comprehensive metabolomic analysis of cells of 3D and 2D cultures	The cells of 3D culture had significantly higher metabolites levels than those of the 2D culture	[121]
BC cell lines (RT4 and PDX)	3D spheroids of RT4 cells were generated using 96-well micro honeycomb plates (1 × 10^4^ cells/well with 2% of Matrigel); 3D spheroids of PDX cells were generated using 96-well low attachment plates (3.9 × 10^4^ cells/well, without Matrigel)	Chemosensitizing effect of glycoalkaloids with cisplatin in RT4 and PDX cells using 2D and 3D cell culture models.	Significantly higher IC_50_ values in cells of 3D cultures than those of 2D monolayers of both RT4 and PDX.	[122]

## Concluding remarks and future perspectives

The present article discussed different types of BC, current practices for its management, and importance of 3D culture systems for screening and evaluation of new cancer therapeutics. Development of appropriate 3D culture models mimicking the in vivo tumorigenesis microenvironment will enable to better addressing of the key steps during cancer formation, growth, and metastasis. Although being more advantageous than 2D culture systems in terms of cell-cell contact and survival, the applicability of 3D culture models is challenged by the fewer number of published studies as well as the cost and complexity of culture conditions. Future studies are still required to overcome these challenges. These studies will definitely help to effectively screen a large number of drugs to be used for the treatment of BC.

## Data Availability

All data are available from the corresponding author upon request.
